# Multivariate Analysis of Risk Factors of Cerebral Infarction in 439 Patients Undergoing Thoracic Endovascular Aneurysm Repair

**DOI:** 10.1097/MD.0000000000003335

**Published:** 2016-04-18

**Authors:** Yuji Kanaoka, Takao Ohki, Koji Maeda, Takeshi Baba, Tetsuji Fujita

**Affiliations:** From the Division of Vascular Surgery, Department of Surgery, Jikei University school of Medicine, Tokyo, Japan.

## Abstract

The aim of the study is to identify the potential risk factors of cerebral infarction associated with thoracic endovascular aneurysm repair (TEVAR).

TEVAR was developed as a less invasive surgical alternative to conventional open repair for thoracic aortic aneurysm treatment. However, outcomes following TEVAR of aortic and distal arch aneurysms remain suboptimal. Cerebral infarction is a major concern during the perioperative period.

We included 439 patients who underwent TEVAR of aortic aneurysms at a high-volume teaching hospital between July 2006 and June 2013. Univariate and multivariate logistic regression analyses were performed to identify perioperative cerebral infarction risk factors.

Four patients (0.9%) died within 30 days of TEVAR; 17 (3.9%) developed cerebral infarction. In univariate analysis, history of ischemic heart disease and cerebral infarction and concomitant cerebrovascular disease were significantly associated with cerebral infarction. “Shaggy aorta” presence, left subclavian artery coverage, carotid artery debranching, and pull-through wire use were identified as independent risk factors of cerebral infarction. In multivariate analysis, history of ischemic heart disease (odds ratio [OR] 6.49, *P* = 0.046) and cerebral infarction (OR 43.74, *P* = 0.031), “shaggy aorta” (OR 30.32, *P* < 0.001), pull-through wire use during surgery (OR 7.196, *P* = 0.014), and intraoperative blood loss ≥800 mL (OR 24.31, *P* = 0.017) were found to be independent risk factors of cerebral infarction.

This study identified patient- and procedure-related risk factors of cerebral infarction following TEVAR. These results indicate that patient outcomes could be improved through the identification and management of procedure-related risk factors.

## INTRODUCTION

Thoracic endovascular aneurysm repair (TEVAR) was developed as a minimally invasive surgical procedure for the treatment of thoracic aortic aneurysm (TAA).^1–4^ Adverse neurological events are reported less frequently following TEVAR than after open repair. In patients with highly angulated aortic arches and presence of neck vessels, complete aneurysm repair with TEVAR alone may be technically challenging.^5^ Combining TEVAR with the debranching of neck vessels can broaden the applications of TEVAR; however, cerebral infarction remains a considerable concern during the perioperative period with the use of this approach.^4–17^ According to a review of 7 studies, including a total of 1,394 patients who underwent TEVAR, the incidence of cerebral infarction following TEVAR ranges from 2.3% to 8.2%.^18^ The reported mortality rate associated with cerebral infarction following TEVAR was 20% in a study of 139 patients^19^ and 33% in a separate study.^18^ Increased understanding of patient-related risk factors of cerebral infarction would inform preoperative risk stratification and clarify surgery-related modifiable risk factors leading to improved patient outcomes. Previously reported risk factors of cerebral infarction following TEVAR include involvement of the proximal descending aorta,^18^ high-grade atheroma of the aortic arch,^18^ history of stroke,^18^ obesity,^20^ greater intraoperative blood loss,^20^ female sex,^21^ longer surgery duration,^21^ and occlusion of the left subclavian artery.^21,22^ There may be many more risk factors that are yet to be identified. The aim of the present study was to identify the potential risk factors of cerebral infarction associated with TEVAR.

## METHODS

### Study Design

A retrospectively collected database of patients undergoing TEVAR at our institution from June 2006 to June 2013 was used in the present study. We collected data from medical charts and reviewed patient characteristics, comorbidities, 30-day mortality. The Review Board at our institution waived the requirement for informed consent. Patient characteristics, comorbidities, 30-day mortality, causes of death, and perioperative cerebral infarction were comprehensively evaluated. Elective TEVAR procedures were performed in 439 patients between June 2006 and June 2013. The inclusion criteria were consecutive patients who underwent TEVAR for fusiform descending TAA > 55 mm or saccular TAA, and fusiform aortic arch aneurysms > 60 mm or saccular arch aneurysm considered unfit for open repair because of severe comorbidities. The exclusion criteria were patients who underwent surgery for acute or chronic aortic dissection and those who underwent emergent surgery. In total, 202 and 237 cases of descending thoracic aortic and distal arch aneurysms, respectively, were included in the present study. Of the 439 patients, 112 required neck vessel reconstruction. In cases where the proximal neck could not be sufficiently secured because of arcuate or distal arch aneurysms, additional procedures, such as neck vessel debranching, were performed. Since 2008, totally endovascular TEVAR with the chimney technique, which involves the use of a chimney stent to secure carotid artery blood flow, has been implemented at our institution. The retrograde in-situ branched stent graft (RIBS) technique, in which stent grafts are fenestrated and branched, and branched TEVAR using custom-made branched stent grafts (the Zenith Branched Thoracic Arch Graft, hereafter, “a-Branch”) were also performed in certain cases during the study period.

### Imaging

Contrast-enhanced computed tomography (CT) was performed as a preoperative assessment in all cases. We transferred Digital Imaging and Communications in Medicine data of the preoperative CTA to the Aquarius net station (TeraRecon Inc, Foster City, CA) to create 3-dimensional image reconstructions. Access routes and aneurysmal pathology were evaluated by preoperative contrast-enhanced CT. In order to optimize protocols for individual patients, treatment strategy regarding device selection and surgical approach were determined on the basis of preoperative discussions. Fundamentally, we attempted to reach favorable proximal or distal aortic necks with a minimum distance of 15 mm. In cases with highly angulated aortic arches, we aimed to secure proximal landing zones at a distance of 20 mm. In cases where the left subclavian artery was completely covered, we evaluated the communication between the left and right vertebral arteries using CT.

### Surgical Procedure

All TEVAR was performed in the hybrid operation room using Innova (GE Healthcare UK Ltd, Buckinghamshire, England) and Artis zeego (Siemens Medical Solutions Inc, PA); TEVAR procedures were performed under general anesthesia. Whenever possible, the proximal landing zone of ≥20 mm in length was required. Three attending surgeons performed the procedures.

### TEVAR With Left Subclavian Artery Coverage

Where the proximal landing zone of ≥20 mm in length is not possible, we planned to cover the left subclavian artery and deploy stent grafting. In these cases, however, we first confirmed communication between both sides of the vertebral artery. Where insufficient communication was identified, or if the left internal thoracic artery was used for bypass, we considered the use of a carotid–subclavian bypass. In cases where the left subclavian artery was completely covered, we evaluated the communication between the left and right vertebral arteries using intraoperative angiography. After covering the left subclavian artery, we confirmed the patency of the posterior inferior cerebellar artery and both sides of the basilar artery by introducing contrast into the right vertebral artery. The subclavian steal phenomenon was assessed by observing the flow through the left vertebral artery and left subclavian artery.

**FIGURE 1:**
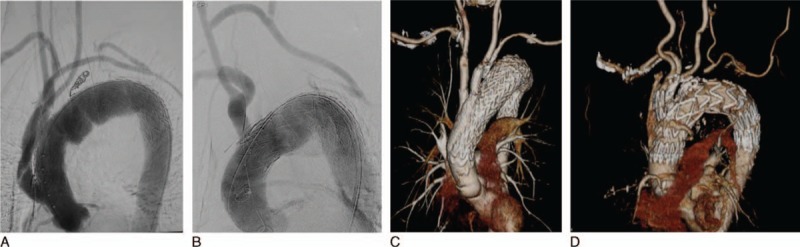
(A) Total debranching TEVAR. (B) TEVAR following carotid–carotid–left subclavian artery bypass. (C) 3D image following chimney TEVAR, (D) 3D image following TEVAR with a double-inner-branch stent graft. TEVAR = thoracic endovascular aneurysm repair.

**FIGURE 2:**
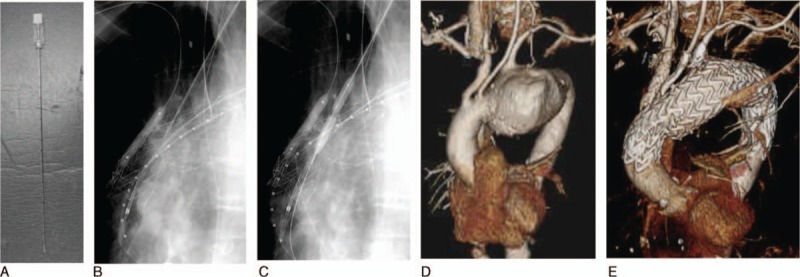
Retrograde in-situ stent graft (RIBS) technique. (A) A 18G PTGBD needle for RIBS, (B) a thoracic stent graft is punctured by a PTGBD needle after the deployment of a stent graft. (C) The puncture site is dilated using a PTA balloon. (D) 3D image before TEVAR. (E) 3D image after TEVAR. PTA = percutaneous transluminal angioplasty, PTGBD = percutaneous transhepatic gallbladder drainage, RIBS = Retrograde in-situ stent grafting, TEVAR = thoracic endovascular aneurysm repair.

In cases where we were unable to secure a 20-mm proximal neck, even with the left subclavian artery covered, we performed TEVAR following a bypass to the neck vessels from the ascending aorta (Figure 1A) or debranching with a carotid–carotid artery bypass (Figure 1B). The chimney technique was used during debranching TEVAR, in which a chimney stent was placed in the carotid artery (Figure 1C). The RIBS technique was also used, in which the stent graft was introduced and fenestrated from the carotid artery in a retrograde manner after the stent graft had been deployed at the aortic arch with covering neck vessels (Figure 2).

### Total Debranching TEVAR

When debranching from the ascending aorta, we performed median sternotomy before partially clamping the ascending aorta. The proximal site of a 10-mm prosthetic graft (Gore-Tex; W. L. Gore and Associates, Flagstaff, AZ) was anastomosed to the ascending aorta, and the distal site of the graft was anastomosed to the brachiocephalic artery, left common carotid artery, and left subclavian artery. Simple clamping was performed when neck vessels were anastomosed. On the passage of stent grafts through the aortic arch during TEVAR, we clamped the graft to prevent cerebral embolization.

### TEVAR With Carotid-Carotid Bypass

The common carotid artery was exposed during bypass procedures using a skin incision ∼3 cm in length along the anterior border of the sternomastoid muscle. Carotid arteries were anastomosed under simple clamping using ringed polytetrafluoroethelyne (ePTFE) vascular grafts (Gore-Tex 6–8 mm; W. L. Gore and Associates) and passed posterior to the esophagus via the shortest route possible. As shown, when stent grafts were passed through the aortic arch, the proximal site of the right common carotid artery was clamped and the left subclavian artery was occluded with a balloon in order to prevent embolism.

### Chimney TEVAR

In the chimney technique, common carotid arteries were exposed and a sheath was inserted in a retrograde manner before stents or stent grafts were used to secure blood flow to carotid arteries.

Bare stents were used for left common carotid arteries and were deployed as stent grafts from the proximal side of the left carotid artery in cases where the left common carotid artery could be covered and the aneurysmal neck could be sufficiently secured. Further, when deploying from zone 0, an Excluder leg or leg extension (W.L. Gore and Associates) was used for brachiocephalic and carotid arteries. Chimney stents were deployed from the proximal ascending aorta whenever possible in order to minimize gutter endoleaks (Figure 1C).

### Retrograde In-Situ Branched Stent Graft (RIBS) Technique

In the RIBS technique, we exposed the carotid artery in the same manner as in the chimney technique. A sheath was inserted in a retrograde manner before the deployment of a thoracic stent graft from the ascending aorta to the thoracic descending aorta. Thereafter, the thoracic stent graft was punctured with an 18-G percutaneous transhepatic gallbladder drainage (PTGBD) needle from the carotid artery sheath. This technique allows the insertion of a guide wire from the PTGBD needle once the thoracic stent graft has been accessed. A covered stent was deployed after the dilation of the aperture using a high-pressure percutaneous transluminal angioplasty (PTA) balloon, for example, a 6–20 mm Conquest PTA dilation catheter (BARD International, Inc, Murray Hill, NJ; Figure 2). We used iCAST™-covered stents (Atrium Medical Corporation, Hudson, NH) and Fluency stent grafts (BARD International, Inc, Murray Hill, NJ) throughout the study period. All these techniques were introduced after receiving approval from the Institutional Ethical Committee. Informed consent was provided by all study participants.

### Branched TEVAR With a-Branch

Branched TEVAR with a-Branch, that is, double-inner-branch stent grafts, was originally designed with fenestrations for stenting of the brachiocephalic and carotid arteries and includes sleeves in fenestrated sections to reduce the development of type III endoleaks. Double-inner-branch stent grafts (a-Branch) were deployed using radiopaque markers on fenestrated sections as guides before branching from carotid arteries.

### TEVAR Procedure and Embolic Protection During TEVAR

**FIGURE 3:**
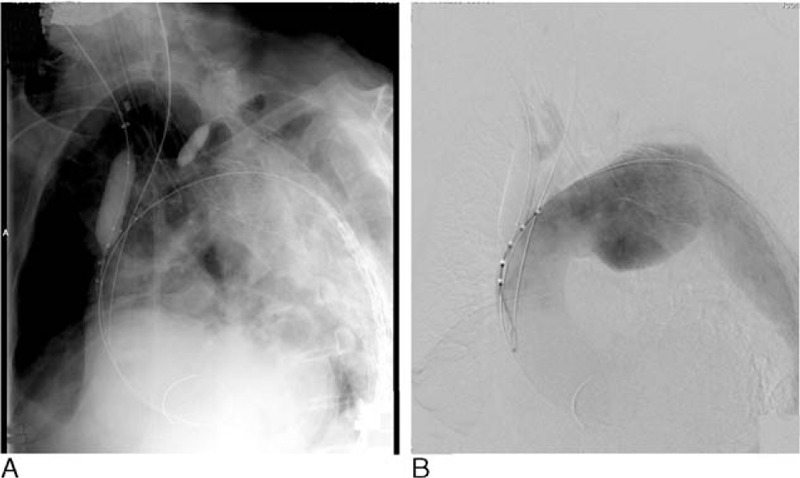
(A) Stroke prevention technique using balloon occlusion and clamping of neck branches. (B) Aortography confirming stagnant neck blood flow.

The left subclavian artery was occluded with a 10-mm balloon during the procedure at the time when stent grafts passed through the aortic arch. Carotid arteries were then directly and manually clamped bilaterally to prevent cerebral embolism (Figure 3). Stent grafts were inserted unilaterally into the common femoral or iliac artery through a stiff wire. In patients with extremely tortuous aortae, guide wires advanced from the right upper extremity were caught using a snare catheter inserted from the cut-down side. Stent grafts were then inserted using the pull-through technique. The following types of devices were used: TAG (including the C-TAG; W.L. Gore; and Associates), Talent (Medtronic Vascular, Santa Rosa, CA), Valiant (Medtronic Vascular), and TX-2 (Cook Inc, Bloomington, IN).

### Endopoints and Cerebral infarction

Postoperative stroke was the primary endpoint of this analysis. The diagnosis of cerebral infarction was based on clinical signs and symptoms indicating new neurologic deficit lasting >24 hour and was confirmed by magnetic resonance imaging (MRI), including diffusion-weighted imaging. The modified Rankin Scale was used to classify disabilities after cerebral infarction. The follow-up period was until the patients were discharged from hospital.

### Follow-Up

These patients were followed up after ∼1, 6, and 12 months postoperatively and annually thereafter in uneventful cases after TEVAR. The follow-up consisted of physical examination, blood tests, radiograph, and contrast-enhanced CT. Endoleaks, aneurysm shrinkage, or enlargement were evaluated by contrast-enhanced CT.

### Statistical Analyses

Univariate analyses were performed to identify patient-related and procedure-related risk factors for stroke during TEVAR. To establish the significance of differences between the means of the different measurements obtained from patients who did or did not have cerebral infarction, Student's *t* test was used, when continuous data exhibited a normal distribution. For comparison of nonparametric data, the Mann–Whitney *U* test was used. Categorical data in the 2 groups were evaluated using the chi square or Fisher's exact test. Each continuous variable was then correlated with the development of stroke by univariate chi square test to select a single cutoff point that maximized significance but preserved clinical utility. Then, only factors that achieved a level of *P* < 0.05 after univariate testing were entered in multiple logistic regression analysis as categorical variables with the 2 categories. IBM SPSS v20.0 (NY) was used for statistical analyses. Patient-related factors included in univariate analysis were as follows: sex, age, aneurysm size, diabetes, dyslipidemia, ischemic heart disease, cerebrovascular disease, chronic kidney disease (CKD), malignancy, chronic obstructive pulmonary disease (COPD), atrial fibrillation, smoking, history of cerebral infarction, preoperative “shaggy aorta,” and current use of antiplatelet and/or anticoagulant agents. CKD was defined as an estimated glomerular filtration rate of <45 mL/min/1.73 m^2^. COPD was defined as a forced expiratory volume in 1 s (FEV 1.0) of <700 mL and/or requirement of home oxygen therapy with emphysematous changes in chest CT. “Shaggy aorta” was defined as the presence of a mural thrombus in a normal nonaneurysmal aorta with a circumference >3/4 of the circumference of a normal aorta, a thickness of ≥5 mm, and a length of ≥2.5 cm on preoperative CT. Mural thrombi associated with aneurysms were excluded from our definition of “shaggy aorta.” However, we did not consider mural thrombus of the ascending aorta to be suitable for TEVAR. Procedure-related factors included in univariate analysis were as follows: surgery duration, intraoperative blood loss volume, fluoroscopy time, contrast medium dose, blood transfusion volume, use of pull-through wires, type of device deployed, landing zone, carotid debranching, and complete coverage of the left subclavian artery. In addition, we assessed the association between endoleaks, shrinkage, and expansion of aneurysmal sacs with cerebral infarction. Aneurysmal sac shrinkage was defined as a reduction of ≥5 mm in the maximum short axis diameter during the follow-up period. Sac expansion was defined as an increase of ≥5 mm in the maximum short axis diameter during the follow-up period.

## RESULTS

A total of 333 (75.9%) male and 106 (24.1%) female patients with a mean age of 74.0 years were included in the present study. The mean maximum TAA short axis was 63.6 ± 13.7 mm. All 202 cases of descending thoracic aortic aneurysm were successfully managed with TEVAR only. The left subclavian artery was completely covered using TEVAR alone in 125 out of 237 patients. Of the 112 patients who received neck vessel reconstruction, total arch replacement using the “elephant trunk” procedure was performed in 8 patients with concurrent dilated ascending aortae prior to TEVAR. Debranching TEVAR and total debranching from the ascending aorta were performed in 40 and 11 patients, respectively. Carotid–carotid artery crossover bypass was performed in 29 patients. Of these 29 patients, stent grafting in the brachiocephalic artery with implantation into zone 0 was performed in 4 patients. Chimney TEVAR was performed in 52 patients. Of these patients, stent grafting in the carotid artery with implantation into zone 0 was performed in 30 patients and bare stenting of the carotid artery with implantation into zone 1 was performed in 22 patients. Further, we used branched stent grafts in 13 patients by using the RIBS procedure with fenestrated stent grafts in 11 patients and double-inner-branch custom-made stent grafts in 2 patients.

Four patients (0.9%) died of perioperative complications due to myocardial infarction, sudden death, ascending aortic dissection, and multiple organ failure (MOF) due to visceral embolism including superior mesenteric artery. Cerebral infarctions were identified in 17 patients (3.9%); these involved anterior circulation stroke in 14 cases (82.4%), posterior circulation stroke in 1 case, and both anterior and posterior in remaining 2 cases. Moreover, all 17 cases were diagnosed with cerebral infarction within 48 hours after the procedure. All 17 strokes were diagnosed as embolic strokes based on MRI findings. According to the modified ranking scale, 1 patient was scale 1, 3 patients were scale 2, 3 patients were scale 3, 6 patients were scale 4, and 4 were scale 5. Postoperatively, all the patients suffering cerebral infarction survived. Postoperatively, all cases underwent follow-up observations, and the mean observation period was 23.9 months (1–84 months).

In univariate analysis, history of coronary heart disease, history of cerebral infarction, concomitant cerebral vascular disease, and “shaggy aorta” identified on preoperative CT were found to be significantly associated with cerebral infarction (Table 1). Left subclavian artery coverage, carotid artery debranching, use of a pull-through wire during surgery, surgery duration ≥240 minutes, and intraoperative blood loss volume ≥250 mL were found to be correlated with cerebral infarction (Tables 1 and 2). History of coronary heart disease, “shaggy aorta,” use of a pull-through wire during surgery, and intraoperative blood loss volume ≥800 mL remained significantly associated with cerebral infarction in multivariate analysis (Table 3).

**TABLE 1:**
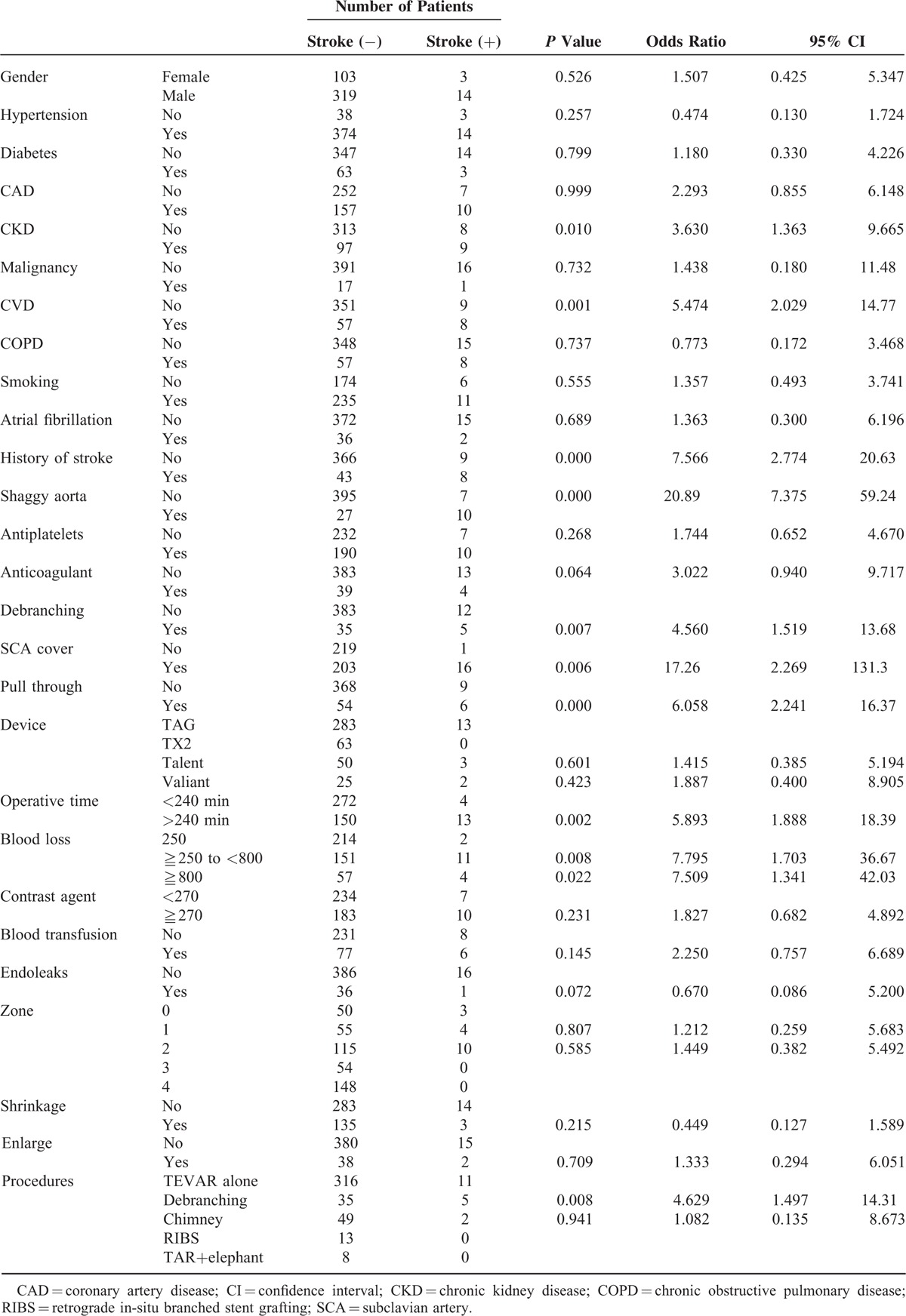
Risk Factors of Perioperative Stroke

**TABLE 2:**
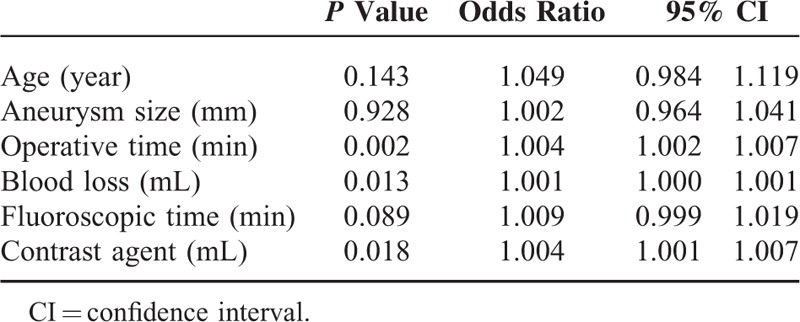
Risk Factors of Perioperative Stroke: Continuous Numeric Variables

**TABLE 3:**
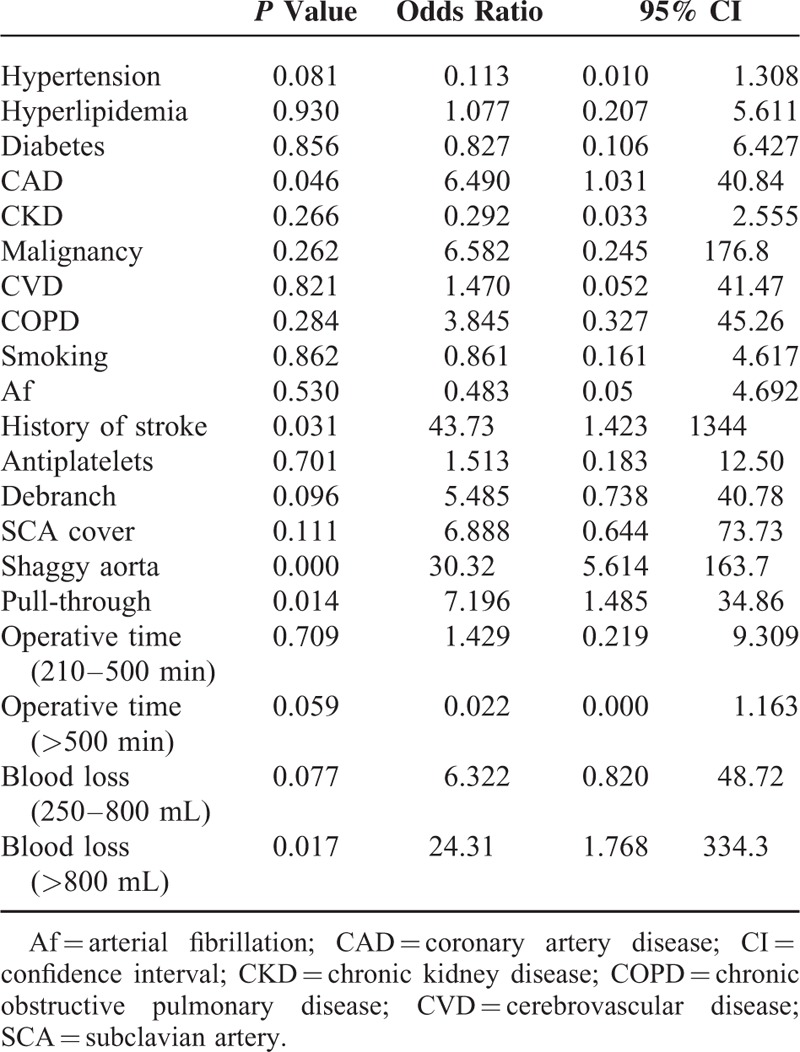
Adjusted Odds Ratio of Risk Factors of Perioperative Stroke

## DISCUSSION

Cerebral infarction is the leading cause of neurologic deficit following TEVAR and is associated with considerable risk of operative mortality. Therefore, the identification of patient-related risk factors of cerebral infarction may provide information regarding risk stratification strategies for TEVAR. Further, the identification of procedure- and surgery-related risk factors may improve patient outcomes. The stroke incidence during TEVAR has been reported to range from 2.3% to 8.2%. Our findings of 3.9% were consistent with previous reports. In the literatures, proximal descending aorta,^18^ prior stroke,^18^ high-grade atheroma of the aortic arch,^19^ proximal landing zone,^19,23^ obesity,^20^ greater intraoperative blood loss,^20^ female sex,^21^ longer duration of the procedure,^21^ and left subclavian coverage^21,22,24^ were the risk factors of cerebral infarction. On the other hand, a previous study indicated that silent postoperative cerebral infarction is frequently overlooked without any symptoms.^6^

Patient-related factors identified in our study as risk factors of cerebral infarction following TEVAR included history of coronary heart disease, history of cerebral vascular disease, and history of cerebral infarction, a particularly strong risk factor. Preoperative “shaggy aorta” was also identified as an independent risk factor of cerebral infarction. Aortae affected by “shaggy aorta” are vulnerable to surgical trauma during surgical procedures, potentially leading to an increased risk of dispersal of multiple emboli into the anterior and posterior circulation and consequent cerebral infarction. The patient-related factors identified in our study as risk factors of cerebral infarction following TEVAR were consistent with previous reports. A history of cerebral infarction indicated the presence of atheroma in the aortic arch or neck branches. Guidewires and stent grafts placed in the aortic arch have the potential to scatter the atheroma in the aortic arch or neck branches. When TEVAR is performed for patients with a history of cerebral infarction, the use of a pull-through wire should be avoided.

Procedure-associated risk factors of cerebral infarction identified in our study included debranching procedures, left subclavian artery coverage, and the use of pull-through wires. Pull-through wires are known to abrade the brachiocephalic artery trunk and aortic arch, potentially increasing the risk of micro emboli formation and resultant cerebral infarction. In particular, when we perform TEVAR for the patients with patient-related risk factors including history of cerebral infarction and “shaggy aorta,” these debranching TEVAR and pull-through wire should be avoided. Device types were not found to be associated with risk of cerebral infarction, whereas increased surgery duration, increased intraoperative blood loss volume, and increased contrast medium dose were identified as risk factors of cerebral infarction. These findings are likely because of the association between debranching procedures and increased surgery duration and intraoperative blood loss volumes. When cases with intraoperative blood loss volumes ≥800 mL were excluded from the multivariate analysis, none of these factors remained statistically significant. When we evaluated each surgical method, we found that cerebral infarction was less frequent after TEVAR involving total arch replacement and the “elephant trunk” procedure than in debranching TEVAR. Therefore, totally endovascular TEVAR including the chimney technique, the RIBS technique, or with branched devices including a-Branch appears to be more appropriate in patients at risk of cerebral infarction. As intraoperative embolization is considered a major cause of cerebral infarction,^23^ we performed balloon occlusion of the left subclavian artery and manual compression and blockage of the carotid artery when stent grafts were passed through the aortic arch (Figure 3). When performing debranching, we clamped bypass grafts from the ascending aorta at the point stent grafts were passing through the aortic arch to prevent embolization. When bypassing the carotid arteries, we clamped the proximal right common carotid artery to prevent embolization. Given the use of these protective procedures against cerebral infarction, cerebral infarction may have been more frequent following debranching TEVAR because of the development of emboli during clamping of the ascending aorta or carotid artery during the debranching procedure. Cerebral infarction occurred in 3.5% of patients who underwent TEVAR alone without neck vessel reconstruction. Aortic arch stent landings were found to be associated with increased risk of cerebral infarction as cerebral infarction was identified in 8.7% of patients with stents implanted into zone 2. Cerebral infarction occurred more often in cases where debranching was performed: 4 out of 29 patients (13.8%) that underwent TEVAR with carotid–carotid artery crossover bypass and 1 out of 11 (9.1%) patients that underwent total debranching from the ascending aorta were found to have cerebral infarctions. Generally, bypass procedures between the carotid arteries and aortic arch implantation sites do not decrease the risk of cerebral infarction. Although the difference was not found to be statistically significant, fewer patients with stent implantation into zone 0 had cerebral infarctions than patients with stent implantation into zones 1 or 2. Cerebral infarctions were identified in 3 out of 50 (6%) patients that had stents implanted into zone 0. Notably, 2 of the 3 cases of cerebral infarction occurred following debranching TEVAR. Of the 42 patients who received totally endovascular procedures where the ascending aorta landing was performed using the chimney or RIBS technique, only 1 (2.4%) patient developed cerebral infarction (Table 4). These findings indicate that totally endovascular techniques, which involve stent grafting of the carotid artery, are the most protective against cerebral infarction. Left occlusion before stent graft deployment is prevented because intraoperative embolization by guidewires or catheters manipulation is considered a major cause of cerebral infarction.^23^ All neck branches including left subclavian artery are occluded by directly and manually clamping or balloon occlusion at the time when stent grafts are passed through the aortic arch during the totally endovascular technique. We can flush out debris before the sheaths inserted via carotid arteries are removed. The potential risk of cerebral infarction during totally endovascular technique may be extremely low. Although we presently utilize the chimney technique with commercially available devices, endoleaks from the gutter represent the greatest technical challenge with this technique. Consequently, completely refined surgical methods are yet to be developed.^16^ We plan to change to gutterless branched stent grafts, such as the double-inner-branch stent graft, in the future.^25^ However, after totally endovascular TEVAR the future approaches to cardiac surgery may be difficult. The percentage of patients developing cerebral infarction following total arch replacement is reportedly ∼4%.^26,27^ Short-term surgical outcomes following TEVAR, such as the incidence of cerebral infarction, could be further improved with the development of more efficacious techniques and the identification of predictors of poor outcome.

**TABLE 4:**
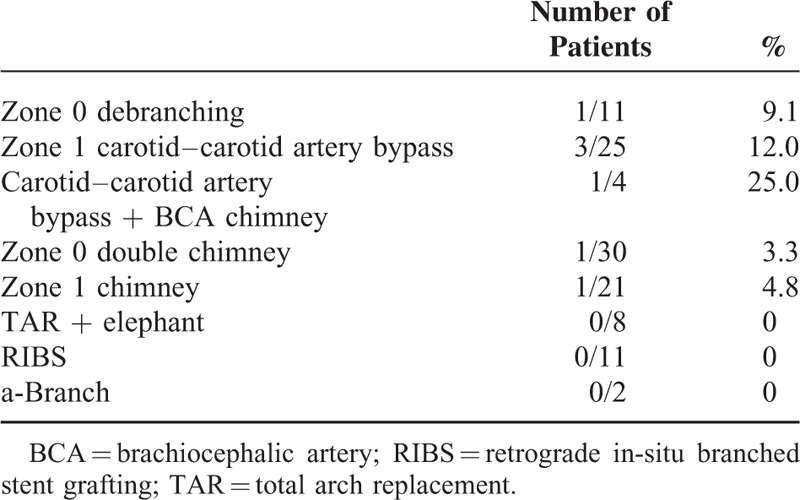
Frequency of Perioperative Stroke According to the Surgical Technique

### Limitations

This retrospective study has several limitations, including potential selection bias and restricted external validity associated with it being a single center analysis. For example, the devices used were selected as per the preferences of the surgeon. The operative procedures varied from simple TEVAR to hybrid TEVAR; therefore, stroke rate might be biased by the learning curve. In addition, the rate of postoperative stroke was lower than expected, which might affect the results of the multivariate analyses including many potential risk factors of cerebral infarction. Furthermore, the effect of the risk factors identified in our study on long-term outcomes of patients is unknown.

## CONCLUSIONS

In multivariate analysis, history of ischemic heart disease and cerebral infarction, “shaggy aorta,” use of a pull-through wire during surgery, and intraoperative blood loss ≥800 mL were found to be independent risk factors of cerebral infarction following TEVAR. Present totally endovascular TEVAR approaches, such as the chimney and the RIBS technique, are superior to debranching TEVAR. When we perform TEVAR for the patients with patient-related risk factors including history of cerebral infarction and “shaggy aorta,” debranching TEVAR and pull-through wire during TEVAR should be avoided. TEVAR may not be the optimal procedure in cases where total arch replacement can be safely performed. The risk of cerebral infarction following TEVAR could be decreased with efforts to reduce surgical complications in all cases.
